# Spontaneous Breathing Trial Techniques for Extubating Adults and Children Who Are Critically Ill: A Systematic Review and Meta-Analysis

**DOI:** 10.1001/jamanetworkopen.2023.56794

**Published:** 2024-02-23

**Authors:** Karen E. A. Burns, Jeena Khan, Vorakamol Phoophiboon, Vatsal Trivedi, J. Carolina Gomez-Builes, Benedetta Giammarioli, Kimberley Lewis, Dipayan Chaudhuri, Kairavi Desai, Jan O. Friedrich

**Affiliations:** 1 Department of Critical Care, St. Michael’s Hospital, Unity Health Toronto, Toronto, Ontario, Canada; 2 Division of General Internal Medicine, Department of Medicine, St. Michael’s Hospital, Unity Health Toronto, Toronto, Ontario, Canada; 3 Interdepartmental Division of Critical Care Medicine, University of Toronto, Toronto, Ontario, Canada; 4 Li Ka Shing Knowledge Institute, St Michael’s Hospital, Unity Health Toronto, Toronto, Ontario, Canada; 5 Department of Health Research Methods, Evidence, and Impact, Faculty of Health Sciences, McMaster University, Hamilton, Ontario, Canada; 6 Royal College of Surgeons in Ireland, University of Medicine and Health Sciences, Dublin, Ireland; 7 Division of Critical Care Medicine, Department of Medicine, Faculty of Medicine, Chulalongkorn University, Bangkok, Thailand; 8 Department of Anesthesiology and Pain Medicine, University of Toronto, Toronto, Ontario, Canada; 9 Institute for Better Health, Trillium Health Partners, Mississauga, Ontario, Canada; 10 Department of Medicine, Faculty of Health Sciences, McMaster University, Hamilton, Ontario, Canada; 11 Department of Critical Care, St. Joseph’s Healthcare, Hamilton, Ontario, Canada; 12 Michael G. DeGroote School of Medicine, Faculty of Health Sciences, McMaster University, Hamilton, Ontario, Canada

## Abstract

**Importance:**

Considerable controversy exists regarding the best spontaneous breathing trial (SBT) technique to use.

**Objective:**

To summarize trials comparing alternative SBTs.

**Data Sources:**

Several databases (MEDLINE [from inception to February 2023], the Cochrane Central Register of Controlled Trials [in February 2023], and Embase [from inception to February 2023] and 5 conference proceedings (from January 1990 to April 2023) were searched in this systematic review and meta-analysis.

**Study Selection:**

Randomized trials directly comparing SBT techniques in critically ill adults or children and reporting at least 1 clinical outcome were selected.

**Data Extraction and Synthesis:**

Paired reviewers independently screened citations, abstracted data, and assessed quality for the systematic review and meta-analysis using Preferred Reporting Items for Systematic Reviews and Meta-analyses (PRISMA guidelines). Data were pooled using random-effects models.

**Main Outcomes and Measures:**

Primary outcomes included SBT success, extubation success, and reintubation.

**Results:**

The systematic review and meta-analysis identified 40 trials that included 6716 patients. Low-quality evidence (14 trials [n = 4459]) suggested that patients were not more likely to pass a pressure support (PS) compared with a T-piece SBT (risk ratio [RR], 1.04; 95% CI, 0.97-1.11; *P* = .31; *I*^2^ = 73%), unless 1 outlier trial accounting for all heterogeneity was excluded (RR, 1.09; 95% CI, 1.06-1.12; *P* < .001; *I*^2^ = 0% [13 trials; n = 3939]; moderate-quality evidence), but were significantly more likely to be successfully extubated (RR, 1.07; 95% CI, 1.04-1.10; *P* < .001; *I*^2^ = 0%; 16 trials [n = 4462]; moderate-quality evidence). Limited data (5 trials [n = 502]) revealed that patients who underwent automatic tube compensation/continuous positive airway pressure compared with PS SBTs had a significantly higher successful extubation rate (RR, 1.10; 95% CI, 1.00-1.21; *P* = .04; *I*^2^ = 0% [low-quality evidence]). Compared with T-piece SBTs, high-flow oxygen SBTs (3 trials [n = 386]) had significantly higher successful extubation (RR, 1.06; 95% CI, 1.00-1.11; *P* = .04; *I*^2^ = 0%) and lower reintubation (RR, 0.37; 95% CI, 0.21-0.65; *P* = <.001; *I*^2^ = 0% [both low-quality evidence]) rates. Credible subgroup effects were not found.

**Conclusions and Relevance:**

In this systematic review and meta-analysis, the findings suggest that patients undergoing PS compared with T-piece SBTs were more likely to be extubated successfully and more likely to pass an SBT, after exclusion of an outlier trial. Pressure support SBTs were not associated with increased risk of reintubation. Future trials should compare SBT techniques that maximize differences in inspiratory support.

## Key Points

**Question:**

Which spontaneous breathing trial (SBT) technique is associated with more successful SBTs and successful extubations among adults and children who are critically ill?

**Findings:**

In this systematic review and meta-analysis that included 6716 critically ill adults and children, patients undergoing pressure support compared with T-piece SBTs were more likely to be extubated successfully and more likely to pass an SBT if the results of an outlier trial were excluded.

**Meaning:**

These findings suggest that pressure support (vs T-piece) SBTs are associated with more successful extubations without increasing risk of reintubation.

## Teaser

This systematic review and meta-analysis evaluates which spontaneous breathing trial (SBT) technique is associated with more successful SBTs and extubations and risk of reintubations among critically ill adults and children.

## Introduction

Approximately 40% of the time spent on mechanical ventilation is attributed to the weaning process.^1^ Compared with nonprotocolized care, a randomized clinical trial^2^ and a systematic review^3^ suggested that weaning protocols shorten the length of stay (LOS) in intensive care units (ICUs), the duration of mechanical ventilation, and the weaning time. To assess their capacity to breathe spontaneously with little or no support, patients typically undergo a spontaneous breathing trial (SBT).^4^

Spontaneous breathing trials aid clinicians in minimizing the duration of invasive ventilation for patients and stimulate timely discussion regarding readiness for extubation. In their attempts to liberate patients from invasive ventilation, clinicians must trade off the risks associated with delayed extubation compared with a premature and unsuccessful extubation. Various techniques are commonly used to conduct SBTs, such as T-piece, continuous positive airway pressure (CPAP), pressure support (PS) with or without positive end-expiratory pressure (PEEP), automatic tube compensation (ATC; providing flow-dependent ventilator support to overcome the resistance of the endotracheal tube during inspiration and expiration), and more recently, high-flow oxygen (HFO; offering variable levels of CPAP depending on the flow rate used). Whereas some SBT techniques such as PS and ATC deliver assistance during inspiration to overcome the resistance of the endotracheal tube, others (eg, CPAP and T-piece) offer no inspiratory assistance. Concerns exist regarding the potential for the former to over-assist and hence overestimate patients’ ability to breathe independently after extubation.^5^ In contrast, T-piece and CPAP offer no inspiratory assistance and may increase patients’ work of breathing during an SBT, thereby potentially underestimating patients’ ability to breathe spontaneously after extubation.^5^ A 2007 consensus statement supported use of initial SBTs with either T-piece breathing or low levels of PS (5-8 cm H_2_O in adults and ≤10 cm H_2_O in children) with or without PEEP (5 cm H_2_O).^6^

A 2014 Cochrane review of 9 trials in adult patients compared T-piece and PS weaning methods, finding nonsignificant differences in weaning success, reintubation, pneumonia, ICU mortality, and LOS between techniques.^7^ In a subgroup analysis including 4 trials (n = 940), the authors found that patients were significantly more likely to pass a PS (vs T-piece) SBT (risk ratio [RR], 1.09; 95% CI, 1.02-1.17).^7^ The review^7^ focused solely on adult patients and did not directly compare alternative SBT methods but rather approaches to weaning that included an SBT. A 2017 systematic review and meta-analysis directly comparing alternative SBT techniques^8^ and the American College of Chest Physicians/American Thoracic Society guideline support use of PS SBTs (5-8 cm H_2_O).^9,10^ Amidst new evidence, we sought to assess the association of alternative SBT techniques with primary outcomes (SBT success, extubation success, and reintubation) and other clinically important outcomes.

## Methods

### Data Sources

In this systematic review and meta-analysis, we conducted a comprehensive search, using database-specific strategies and without language restrictions across several databases, including MEDLINE (from inception to February 2023), the Cochrane Central Register of Controlled Trials (in February 2023), and Embase (from inception to February 2023), to identify potentially eligible trials. Search terms used for article retrieval included are shown in the eAppendix in Supplement 1. We used the optimally sensitive search strategies of the Cochrane Collaboration for both MEDLINE and Embase.^11,12,13^ Eight authors (J.O.F., V.P., V.T., K.L., B.G., K.D., J.C.G.-B., and D.C.) independently screened citation titles and abstracts and subsequently assessed full-text versions of potentially relevant trials. Three authors (V.P., V.T., and J.O.F.) hand searched conference proceedings of 5 scientific meetings from January 1990 to April 2023 where feasible: American Thoracic Society, American College of Chest Physicians (except 1999 to 2002, which was unavailable), International Symposium on Intensive Care and Emergency Medicine, European Society of Intensive Care Medicine, and Society of Critical Care Medicine. Given the nature of the study, no individual patient–level data were used. All data were obtained from published clinical trials. No specific ethics approval was required. A written protocol was registered in the PROSPERO International Prospective Register of Systematic Reviews (CRD42023453264) guided conduct, and the study followed the Preferred Reporting Items for Systematic Reviews and Meta-analyses (PRISMA) reporting guideline.

### Study Selection

We included randomized or quasi-randomized trials (eg, alternating patients, day of week, medical record number, or some other allocation method in which group assignment was known prior to randomization) that directly compared 2 or more SBT techniques in critically ill adults or children and reported at least 1 initial SBT or extubation outcome (success or failure), reintubation, time to first successful SBT, time to extubation or successful extubation, ventilator-associated pneumonia, ICU or hospital LOS, mortality, postextubation use of noninvasive ventilation and HFO, total duration of ventilation, or adverse events as defined by the authors. We excluded trials that evaluated SBTs as part of a weaning strategy; neonatal patients or those who had already undergone tracheostomy; and trials evaluating automated SBTs (eg, SmartCare [Dräger] and INTELLiVENT [Hamilton Medical]), noninvasive ventilation, and SBT compared with no SBT. Two authors (K.E.A.B. and J.O.F.) independently selected trials that met inclusion criteria and adjudicated disagreements.

### Data Extraction and Quality Assessment

Two unblinded authors (K.E.A.B. and J.O.F.) abstracted data regarding the study characteristics, estimates, and risk of bias (including allocation concealment, randomization, blinded outcomes assessment, selective outcomes reporting, completeness of follow-up, and stopping early for benefit) and recorded data on a standardized form.^14^ For each domain, we appraised risk of bias (yes, unclear, or no). The same 2 authors discussed disagreements and resolved them by consensus.

### Statistical Analysis

Data were pooled across studies using DerSimonian and Laird random-effects models. Descriptive statistics were used to describe the outcomes. For binary outcomes and continuous outcomes, we tabulated RRs with 95% CIs and mean differences with 95% CIs, respectively, using the Mantel-Haenszel method in Review Manager, version 5.4 (Cochrane Collaboration).^15^ Trials were summarized based on the techniques compared (eg, T-piece vs other). To report SBT success, we pooled the initial SBT outcome for trials that conducted more than 1 SBT. We assessed statistical heterogeneity for each outcome using the *I*^2^ index, categorized into intervals of 0% to 40% (potentially negligible), 30% to 60% (moderate), 50% to 90% (significant), and 75% or more (considerable).^16,17^ All *P* values were 2-sided, and *P* < .05 was considered statistically significant.

In an a priori sensitivity analysis, we assessed the association of excluding quasi-randomized trials and explored sources of heterogeneity on summary estimates for SBT and extubation outcomes. In case of substantial heterogeneity, we planned a priori to systematically exclude each trial in a full leave-1-study-out analysis to examine for substantial changes to the pooled heterogeneity and assess whether a single trial accounted for the heterogeneity. In subgroup analyses limited to PS compared with T-piece SBTs, we examined the association of chronic obstructive pulmonary disease (with vs without) and levels of PEEP (≤5 cm H_2_O vs ≥6 cm H_2_O) and PS (≤8 cm H_2_O vs ≥9 cm H_2_O) during SBTs on SBT and extubation outcomes. Across all trials, we compared SBTs with vs without inspiratory assistance (eg, PS or ATC vs T-piece or CPAP) on SBT and extubation outcomes. We assessed subgroup differences using the χ^2^ test.^18^

We used the Grading of Recommendations, Assessment, Development and Evaluation (GRADE) system to evaluate the body of evidence associated with primary outcomes and significant secondary outcomes.^19^ For SBT and extubation outcomes, we assessed for publication bias when at least 10 trials were identified.^20^

## Results

### Trial Identification

**Figure 1.**
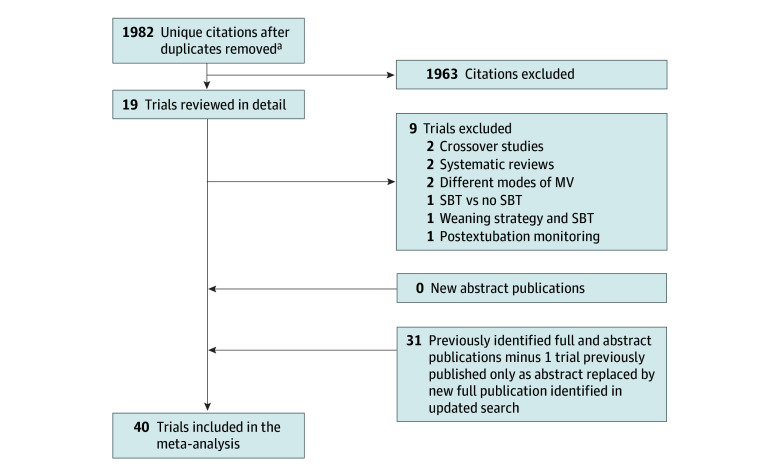
Preferred Reporting Items for Systematic Reviews and Meta-Analyses (PRISMA) Flow Diagram of Trial Selection Of the 40 included trials, 10 were full trial publications, of which 9 were new publications and 1 was a full publication that replaced a previously identified abstract. MV indicates mandatory ventilation; SBT, spontaneous breathing trial. ^a^Included updated search through February 1, 2023, limited to newly indexed citations since prior search.^8^

We identified 1982 new unique citations, of which 1963 were excluded. Of the 19 studies further assessed for eligibility, 9 were excluded^21,22,23,24,25,26,27,28,29^ (Figure 1). In addition to 31 previously included trials,^30,31,32,33,34,35,36,37,38,39,40,41,42,43,44,45,46,47,48,49,50,51,52,53,54,55,56,57,58,59,60^ we identified 10 new full publications,^61,62,63,64,65,66,67,68,69^ including 1 full publication^59^ that replaced a previously included abstract publication. Of the 40 trials included (n = 6797 randomized and n = 6716 patients with outcomes), 9 were new trials (n = 3130 patients analyzed), 6 trials compared 3 SBT techniques^35,36,41,42,60,66^ (but for 1 of the 3-arm trials, the 5-cm H_2_O and 10-cm H_2_O CPAP groups were combined for a single comparison with the third T-piece group^36^), and 1 trial^62^ compared 4 SBT techniques (Table 1). Four trials^33,46,50,52^ from the previous search were published in a foreign language, and 6 trials^37,39,45,48,57,59^ were published as abstracts. All new trials evaluated adults. Three previously included trials^40,55,58^ reported on children. Two trials^50,58^ appeared to be published, at least in part, in duplicate.^70,71^

**Table 1.** Characteristics of Included Trials

Author(s), year (No. randomized)	Interventions	Country	Publication type	Population	Duration of ventilation at inclusion
Feeley et al,^30^ 1975 (25)	T-piece/PEEP (5 cm H_2_O) vs T-piece	US	Full	Adult	Not reported
Hastings et al,^31^ 1980 (18)	IMV/CPAP (5 cm H_2_O) vs T-piece/CPAP (5 cm H_2_O)	US	Full	Adult	Perioperative
Prakash et al,^32^ 1982 (28)	IMV vs SVT (on ventilator)	Netherlands	Full	Adult	Perioperative
Koller et al,^33^ 1983 (45)^a^	CPAP (10 cm H_2_O) vs T-piece/ZEEP	Austria	Full (German)	Adult	Perioperative
Jones et al,^34^ 1991 (106)	CPAP (5 cm H_2_O) vs T-piece/ZEEP	US	Full	Adult	Not reported
Abalos et al,^35^ 1992 (62)	SIMV vs CPAP (4 cm H_2_O) vs T-piece	US	Full	Adult	Perioperative
Bailey et al,^36^ 1995 (82)^a^	T-piece/CPAP (0 cm H_2_O) vs CPAP (5 cm H_2_O) vs CPAP (10 cm H_2_O)	England	Full	Adult	Perioperative
Schinco et al,^37^ 1995 (30)	PS (5 cm H_2_O)/CPAP (5 cm H_2_O) vs CPAP (5 cm H_2_O)	US	Abstract	Adult	Perioperative
Esteban et al,^38^ 1997 (520)^a^	T-piece vs PS (7 cm H_2_O)	Spain and South America	Full	Adult	>48 h
Holanda et al,^39^ 2000 (35)	T-piece vs PS (7 cm H_2_O)	Brazil	Abstract	Adult	>48 h
Farias et al,^40^ 2001 (257)	T-piece vs PS (10 cm H_2_O) ± PEEP (5 cm H_2_O)	Argentina	Full	Pediatric	>48 h
Haberthür et al,^41^ 2002 (90)	PS (5 cm H_2_O)/PEEP (5 cm H_2_O) vs ATC/PEEP (5 cm H_2_O) vs T-piece	Switzerland	Full	Adult	>24 h
Koksal et al,^42^ 2004 (60)	PS (<10 cm H_2_O)/PEEP (<5 cm H_2_O) vs CPAP (<5 cm H_2_O) vs T-piece	Turkey	Full	Adult	>48 h
Matić and Majerić-Kogler,^43^ 2004 (260)	T-piece vs PS (8 cm H_2_O)	Croatia	Full	Adult	>48 h
Cohen et al,^44^ 2006 (99)	ATC/CPAP (5 cm H_2_O) vs CPAP (5 cm H_2_O)^b^	Israel	Full	Adult	>24 h
Liang et al,^45^ 2006 (97)	ATC vs T-piece	Taiwan	Abstract	Adult	>4 d
Colombo et al,^46^ 2007 (120)	T-piece vs PS (7 cm H_2_O)/PEEP (5 cm H_2_O)	Brazil	Full (Portuguese)	Adult	>48 h
Matić et al,^47^ 2007 (136)	T-piece vs PS (not specified)	Croatia	Full	Adult	>24 h
Fayed and El Feky,^48^ 2008 (30)	ATC/CPAP (5 cm H_2_O) vs CPAP (5 cm H_2_O)^b^	Egypt	Abstract	Adult	>24 h
Cohen et al,^49^ 2009 (180)	ATC/CPAP (5 cm H_2_O) vs PS (7 cm H_2_O)/CPAP (5 cm H_2_O)^b^	Israel	Full	Adult	>24 h
Zhang and Qin,^50^ 2009 (208)	T-piece vs PS (5 cm H_2_O)/PEEP (5 cm H_2_O)	China	Full (Chinese)	Adult	Not reported
Figueroa-Casas et al,^51^ 2010 (122)^a^	ATC/PEEP (5 cm H_2_O) vs CPAP (5 cm H_2_O)^b^	US	Full	Adult	>24 h
Molina-Saldarriaga et al,^52^ 2010 (50)	CPAP/PEEP (permitted) vs T-piece/PEEP (permitted)^c^	Colombia	Full (Spanish)	Adult	>48 h
Cekman and Erdemli,^53^ 2011 (40)	CPAP (<5 cm H_2_O) vs T-piece	Turkey	Full	Adult	>48 h
Vats et al,^54^ 2012 (40)	T-piece vs PS (7 cm H_2_O)	India	Full	Adult	Not reported
El-Beleidy et al,^55^ 2013 (47)^a^	ATC/CPAP (5 cm H_2_O) vs PS (6-10 cm H_2_O)/CPAP (5 cm H_2_O)^b^	Egypt	Full	Pediatric	>24 h
Lourenço et al,^56^ 2013 (30)^a^	T-piece vs PS (not specified)	Brazil	Full	Adult	Perioperative
Sherif and Atalaah,^57^ 2013 (100)	PS (not specified) vs PS/ATC	Egypt	Abstract	Adult	Not reported
Selek et al,^61^ 2014 (50)^d^	T-piece (under spontaneous breathing) vs ATC/CPAP (≤5 cm H_2_O)	Turkey	Full (Turkish and English)	Adult	>24 h
Zanfaly,^62^ 2014 (120)^d^	ATC/PEEP (5 cm H_2_O) vs PS (7 cm H_2_O) vs CPAP (5 cm H_2_O) vs T-piece	Egypt	Full	Adult	>48 h
Bilan and Ganji,^58^ 2015 (51)	CPAP vs T-piece	Iran	Full	Pediatric	Not reported
Chittawatanarat et al,^59^ 2018 (520)	T-piece vs PS (7 cm H_2_O)/PEEP (<5 cm H_2_O)	Thailand	Full	Adult	>12 h
Teixeira et al,^60^ 2015 (165)	PS (7 cm H_2_O) vs PAV+ vs T-piece	Brazil	Full	Adult	>24 h
El-Shahat et al,^63^ 2015 (166)^d^	PS/PEEP (0-5 cm H_2_O) vs ATC/PEEP (0-5 cm H_2_O)	Egypt	Full	Adult	>24 h
Santos Pellegrini et al,^64^ 2018 (190)^d^	T-piece vs PS (10 cm H_2_O)	Brazil	Full	Adult	>48 h
Subirà et al,^65^ 2019 (1153)^d^	T-piece (×2 h) vs PS (8 cm H_2_O; 30 min)	Spain	Full	Adult	>24 h
Liu et al,^66^ 2019 (268)^d^	T-piece vs PS (7 cm H_2_O) vs HFO (at end of ETT)	China	Full	Adult	>48 h
Fossat et al,^67^ 2021 (106)^d^	T-piece vs HFO (at end of ETT)	France	Full	Adult	>24 h
Thille et al,^68^ 2022 (983)^a^^,^^d^	PS vs T-piece	France	Full	Adult	>24 h
Lee et al,^69^ 2022 (108)^d^	T-piece vs HFO (at end of ETT)	Korea	Full	Adult	>24 h

Abbreviations: ATC, automatic tube compensation; CPAP, continuous positive airway pressure; ETT, endotracheal tube; HFO, high-flow oxygen; IMV, intermittent mandatory ventilation; PAV+, proportional assist ventilation with load-adjustable gain factors; PEEP, positive end-expiratory pressure; PS, pressure support ventilation; SIMV, synchronized intermittent mandatory ventilation; SVT, spontaneous ventilation trial; ZEEP, zero end-expiratory pressure.

^a^ Lower number of patients analyzed than randomized: Koller et al^33^ (n = 33), Bailey et al^36^ (n = 80), Esteban et al^38^ (n = 484), Figueroa-Casas et al^51^ (n = 118), El-Beleidy et al^55^ (n = 36), Lourenço et al^56^ (n = 28), and Thille et al^68^ (n = 969).

^b^ ATC with 100% compensation.

^c^ CPAP set to 85% of intrinsic PEEP.

^d^ New trials.

### Risk of Bias

Twenty-one trials (52.5%) (5 new^64,65,67,68,69^ and 16 previously included^30,33,34,36,38,40,41,44,49,51,52,53,54,56,59,60^) and 23 trials (57.5%) (6 new^62,64,65,67,68,69^ and 17 previously included^34,36,38,40,41,42,43,44,47,48,49,52,53,54,55,59,60^) were judged to be at low risk of bias for randomization and allocation concealment, respectively. Four trials (10.0%)^30,33,46,51^ were judged to be at high risk of bias with regard to allocation concealment, including 1 quasi-randomized trial^46^ allocating patients based on even or odd days. Only 2 trials (5.0%)^32,66^ assessed outcomes in a blinded manner. Twenty-one trials (52.5%)^30,31,40,41,42,43,44,46,47,48,49,52,53,59,60,62,63,64,65,67,69^ were judged to have complete outcomes reporting, including 6 new trials.^62,63,64,65,67,69^ Finally, 27 trials (67.5%)^30,32,33,36,38,40,41,42,43,44,46,47,48,49,52,53,56,59,60,62,63,64,65,66,67,68,69^ conducted an intention-to-treat analysis, and 33 trials (82.5%)^30,31,33,34,35,36,38,39,40,41,42,43,44,45,46,47,48,49,51,52,53,55,56,59,60,61,62,63,64,65,67,68,69^ did not stop early for benefit. Overall trial quality was moderate (eFigure in Supplement 1).

### Primary Outcomes

#### Initial SBT Success

**Figure 2.**
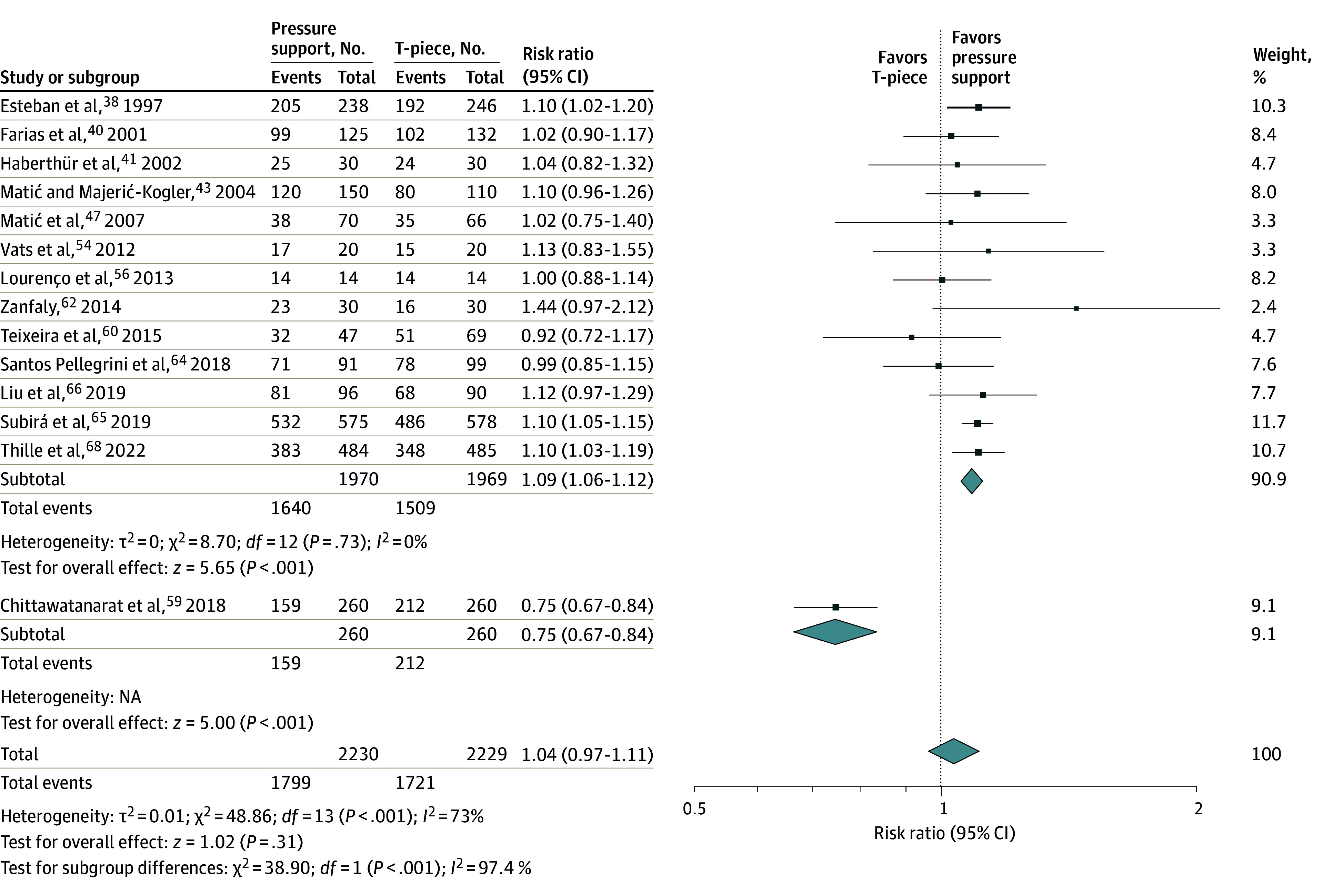
Association of Pressure Support Compared With T-Piece Spontaneous Breathing Trials (SBTs) on SBT Outcome Exclusion of the single pediatric trial (Farias et al^40^) changed the summary estimate to 1.04 (95% CI, 0.96-1.11); *P* = .33; *I*^2^ = 75%. Squares indicate risk ratios, with the size of squares indicating weight; horizontal lines, 95% CIs; and diamonds, overall risk ratios, with outer points of the diamonds indicating 95% CIs. NA indicates not applicable.

Thirty-five trials including 48 SBT comparisons reported initial SBT success including 14 PS vs T-piece,^38,40,41,43,47,54,56,59,60,62,64,65,66,68^ 2 PS and CPAP vs CPAP,^37,62^ 5 PS vs ATC and CPAP,^41,49,55,62,63^ 8 CPAP vs T-piece,^30,33,34,35,36,52,53,62^ 4 ATC vs T-piece,^41,45,61,62^ 3 HFO vs T-piece,^66,67,69^ 4 ATC and CPAP vs CPAP,^44,48,51,62^ 2 intermittent mandatory ventilation (IMV) vs CPAP,^31,35^ and 1 trial each of PS vs HFO,^66^ PS vs proportional assist ventilation with load-adjustable gain factors (PAV+),^60^ PS vs PS and ATC,^57^ T-piece vs PAV+,^60^ IMV vs T-piece,^35^ and IMV vs spontaneous ventilation on the ventilator^32^ (Table 1). Compared with T-piece SBTs, low-quality evidence from 14 trials (n = 4459) supported that patients undergoing PS SBTs were not more likely to pass an SBT (RR, 1.04; 95% CI, 0.97-1.11; *P* = .31) with substantial heterogeneity (*I*^2^ = 73%) (Figure 2 and eTable 1 in Supplement 1).

Low-quality evidence from 4 trials (n = 307) suggests that patients were significantly more likely to pass an ATC and CPAP SBT compared with a CPAP SBT (RR, 1.15; 95% CI, 1.04-1.27; *P* = .004; *I*^2^ = 21%). Similarly, low-quality evidence from 5 trials (n = 502) showed that patients were significantly more likely to pass an ATC and CPAP SBT compared with a PS SBT (RR, 1.12; 95% CI, 1.03-1.21; *P* = .005; *I*^2^ = 0%) (Table 2). Compared with CPAP, low-quality evidence from 2 trials (n = 90) identified that patients were significantly more likely to pass a PS and CPAP SBT (RR, 1.59; 95% CI, 1.05-2.42; *P* = .03; *I*^2^ = 38%).

**Table 2.** Summary Estimates for Comparisons of ATC vs Other Techniques on SBT and Extubation Outcome

Comparison outcome	Trials, No. (N)	Risk ratio (95% CI)	*P* value	*I*^2^, %
**SBT**				
ATC vs T-piece	4 (267)	1.09 (0.88-1.36)	.42	77
ATC/CPAP vs CPAP	4 (307)	1.15 (1.04-1.27)	.004	21
ATC/CPAP vs PS^a^	5 (502)	1.12 (1.03-1.21)	.005	0
ATC/PS vs PS	1 (100)	1.04 (0.94-1.15)	.40	NA
**Extubation**				
ATC vs T-piece	3 (217)	1.23 (0.82-1.86)	.32	76
ATC/CPAP vs CPAP	4 (307)	1.40 (0.84-2.35)	.20	89
ATC/CPAP vs PS^a^	5 (502)	1.10 (1.00-1.21)	.04	0
ATC/PS vs PS	1 (100)	1.07 (0.93-1.23)	.34	NA

Abbreviations: ATC, automatic tube compensation; CPAP, continuous positive airway pressure; NA, not applicable; PS, pressure support; SBT, spontaneous breathing trial.

^a^ Exclusion of the single pediatric trial El-Beleidy et al^55^ did not change the summary estimates of effect for ATC/CPAP compared with PS on initial successful SBT (risk ratio, 1.12 [95% CI, 1.03-1.21]; *P* = .006; *I*^2^ = 0%) and minimally changed the summary estimate for successful extubation (risk ratio, 1.11 [95% CI, 1.01-1.22]; *P* = .03; *I*^2^ = 0%).

#### Extubation Success

**Figure 3.**
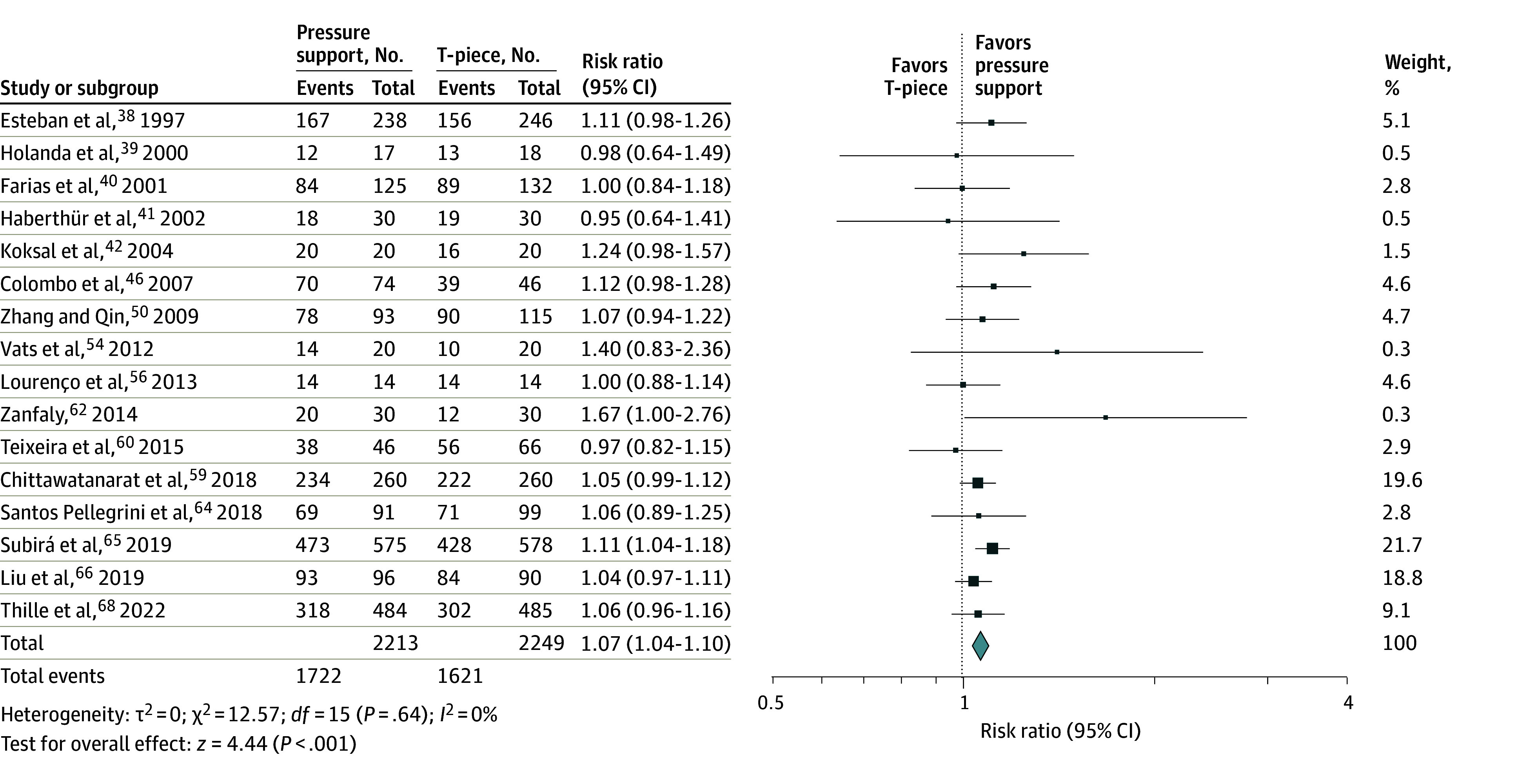
Association of Pressure Support Compared With T-Piece Spontaneous Breathing Trials on Successful Extubation Exclusion of the single pediatric trial (Farias et al^40^) did not change the summary estimate of effect for this outcome. Squares indicate risk ratios, with size of squares indicating weight; horizontal lines, 95% CIs; and diamond, overall risk ratio, with outer points of the diamond indicating 95% CIs.

Thirty-one trials including 44 SBT comparisons reported extubation success, including 16 PS vs T-piece,^38,39,40,41,42,46,50,54,56,59,60,62,64,65,66,68^ 5 PS vs ATC and CPAP,^41,49,55,62,63^ 5 CPAP vs T-piece,^33,34,52,53,62^ 4 ATC and CPAP vs CPAP,^44,48,51,62^ 3 ATC vs T-piece,^41,45,62^ 3 HFO vs T-piece,^66,67,69^ 2 PS vs CPAP,^42,62^ and 1 trial each of IMV vs CPAP,^31^ PS vs HFO,^66^ PS vs PAV+,^60^ PS vs PS and ATC,^57^ T-piece vs PAV+,^60^ and IMV vs a spontaneous ventilation trial^32^ (Table 1). Moderate-quality evidence from 16 trials (n = 4462) supported that patients undergoing PS compared with T-piece SBTs were 7% (95% CI, 4%-10%; number needed to treat, 18 [range, 12-31], assuming baseline risk of successful extubation of 72% [1621 of 2249] from Figure 3) more likely to be extubated successfully (RR, 1.07; 95% CI, 1.04-1.10; *P* < .0001; *I*^2^ = 0%) (Figure 3 and eTable 2 in Supplement 1).

Compared with PS SBTs, low-quality evidence from 5 trials (n = 502) identified that patients undergoing ATC and CPAP SBTs were significantly more likely to be successfully extubated (RR, 1.10; 95% CI, 1.00-1.21; *P* = .04; *I*^2^ = 0) (Table 2). Compared with T-piece, low-quality evidence from 3 trials (n = 386) identified that patients undergoing HFO SBTs were significantly more likely to be successfully extubated (RR, 1.06; 95% CI, 1.00-1.11; *P* = .04; *I*^2^ = 0%).

#### Reintubation Rate

Thirty trials including 41 alternative SBT comparisons reported reintubation, including 13 PS vs T-piece,^38,39,40,42,54,56,59,60,62,64,65,66,68^ 6 CPAP vs T-piece,^34,42,52,53,58,62^ 4 PS vs ATC and CPAP,^49,55,62,63^ 4 ATC and CPAP vs CPAP,^44,48,51,62^ 3 ATC vs T-piece,^45,61,62^ 3 HFO vs T-piece,^66,67,69^ 2 PS vs CPAP,^42,62^ and 1 trial each of IMV vs CPAP,^31^ PS vs HFO,^66^ PS vs PAV+,^60^ PS vs PS and ATC,^57^ T-piece vs PAV+,^60^ and IMV vs a spontaneous ventilation trial^32^ (Table 1). Compared with T-piece SBTs, PS SBTs did not increase reintubation rates (RR, 1.04; 95% CI, 0.90-1.21; *P* = .57; *I*^2^ = 0%).

Patients undergoing HFO (vs T-piece) SBTs with low-quality evidence from 3 trials (n = 386) had significantly lower reintubation rates (RR, 0.37; 95% CI, 0.21-0.65; *P* < .001; *I*^2^ = 0%). One trial (n = 178) comparing PS compared with HFO SBTs had a significantly lower reintubation rate favoring HFO SBTs (RR, 0.29; 95% CI, 0.13-0.68; *P* = .004).

### Secondary Outcomes

There was no association of 1 SBT technique compared with another on ICU mortality, hospital mortality, or the most protracted mortality measure. One trial each reported time to first SBT^64^ and time to successful extubation.^63^ There was no association of the alternative SBT techniques on ICU or hospital LOS and rates of tracheostomy and noninvasive ventilation and HFO use after extubation (eTable 3 in Supplement 1).

### Sensitivity, Subgroup, and Post Hoc Analyses

Excluding 1 trial with opposite SBT and extubation outcomes showed that significantly more patients passed an initial PS (vs T-piece) SBT (RR, 1.09, 95% CI, 1.06–1.12; *P* < .001) without heterogeneity (*I*^2^ = 0%) for moderate-quality evidence from 13 trials (n = 3939). Excluding each of the other trials in a full leave-1-study-out analysis with and without the single outlier trial^59^ did not result in significant changes to the pooled estimate for SBT success (pooled RR range, 1.03-1.04) or heterogeneity (*I*^2^ range, 72%-75%). We did not identify credible subgroup effects in subgroup analyses (eTable 4 in Supplement 1). For PS compared with T-piece comparisons, inspection of funnel plots for 14 trials reporting SBT success and 16 trials reporting extubation success did not suggest publication bias.

## Discussion

In this systematic review and meta-analysis, we identified 40 trials reporting alternative SBT comparisons in 6716 patients including 9 new trials (n = 3130). This nearly doubled the number of included patients, increased precision of summary estimates, and narrowed CIs compared with a prior review and meta-analysis by some members of our group.^8^ Pressure support compared with T-piece was the most commonly investigated SBT comparison. Low-quality evidence showed that patients were not more likely to pass a PS (vs T-piece) SBT with substantial heterogeneity. Exclusion of a single trial^59^ with discordant SBT and extubation outcomes eliminated this heterogeneity and identified that participants were 9% more likely to pass PS (vs T-piece) SBTs. Meta-analysis identified that patients undergoing PS (vs T-piece) SBTs were 7% more likely to be successfully extubated with no increased risk for reintubation. This probability of successful extubation corresponds with a number needed to treat of 18 (95% CI, 12-31). Limited data showed that patients who underwent ATC and CPAP compared with PS SBTs had a significantly higher successful extubation rate. Patients who underwent HFO compared with T-piece SBTs had significantly higher successful extubation and lower reintubation rates. We did not identify credible subgroup effects.

Our systematic review and meta-analysis differs from 2 previous reviews in that we did not include trials that evaluated SBTs as 1 component of a weaning strategy.^7,72^ Contrary to the findings of a 2014 Cochrane review,^7^ we found that patients were only more likely to pass a PS (vs T-piece) SBT after we excluded a single outlier trial^59^ but were significantly more likely to be extubated successfully. Our review also differs from that of Pellegrini et al^72^ in that we included pediatric trials^40,55,58^ and excluded weaning trials.^73,74,75,76^ Similar to both reviews,^7,72^ we did not find that an SBT technique was associated with reintubation or mortality rates.

Considerable debate exists regarding the SBT technique that best approximates patients’ work of breathing after extubation. Clearly an SBT is an imperfect test as it is performed with an endotracheal tube in situ. Furthermore, SBTs do not take into consideration factors that may impact extubation outcome (eg, upper airway resistance, level of consciousness, ability to protect the airway, cough strength, secretions, respiratory muscle weakness and endurance, and cardiac reserve). A meta-analysis of the associations of alternative SBT techniques with physiological outcomes found that metrics of patient effort, including work of breathing (n = 142) and pressure–time product (n = 129), were significantly higher during T-piece (vs PS) SBTs, albeit with considerable heterogeneity (*I*^2^ ≥ 75%).^77^ Moreover, during T-piece (vs PS) SBTs, these physiologic variables were more comparable with the postextubation period, although sample sizes were small, and heterogeneity was moderate (work of breathing: n = 77 [*I*^2^ = 67%]; pressure–time product: n = 52 [*I*^2^ = 62%]); the GRADE system was not used to assess the body of evidence for these outcomes.^77^ These data suggested that T-piece SBTs better reflected the postextubation state. However, the association of these physiologic variables with clinical outcomes in these studies was not assessed.

It is likely that no single SBT technique will be optimal for all intubated patients. Compared with T-piece SBTs, our review suggests that PS SBTs facilitated extubation decision-making for unselected critically ill adults or children. As such, even if PS SBTs underestimate postextubation work of breathing, successful completion of a PS SBT may offset clinician reluctance to extubate, enabling more timely and successful extubation.^78,79^ Importantly, we found that PS SBTs were likely associated with a higher extubation rate without a higher reintubation rate. However, these findings must be interpreted in context. It is likely that many patients in ICUs^8,9^ have a high pretest probability of success and can be extubated easily after an initial SBT.^80^ It is also likely that T-piece SBTs may be appropriate for selected patients (eg, severe left ventricular dysfunction, neuromuscular weakness, or difficult airway). T-piece SBTs may also be preferred when clinicians are uncertain about SBT outcome and want to prioritize having a low false-positive rate for patients passing an SBT and being extubated successfully to limit the risks associated with extubation failure.^81^ However, if T-piece SBTs were used in all patients, including those with a high likelihood of extubation success, they may induce a high false-negative rate. Notwithstanding, reintubation may not only be associated with SBT technique but also with extubation-related factors and coexisting or new illnesses. Furthermore, by disconnecting patients from ventilators, T-piece SBTs do not permit application of PEEP to reduce the potential for loss of lung aeration in the periextubation period. Although passing an initial SBT is an important and commonly reported trial outcome, patients may undergo serial SBTs before being extubated, and trials varied in how often the assigned SBT technique was applied. Regardless, clinicians and patient and family stakeholders prioritize outcomes associated with being successfully extubated over passing an SBT.^82^

### Strengths and Limitations

Our review has strengths. To our knowledge, this is the largest systematic review and meta-analysis comparing SBT trials conducted to date. Our systematic review and meta-analysis was guided by a registered protocol and strengthened by an extensive search, duplicate citation screening and data abstraction, use of random-effects models and the GRADE method, and conduct of prespecified subgroup analyses.

Our review also has important limitations. First, the included trials were of variable methodologic quality. Trials used various criteria to identify SBT candidates and varied in their use of structured daily screening. The absence of daily screening and incorporation of extubation criteria among screening criteria may have delayed initial SBT conduct. Trials also varied in how often the assigned SBT technique was applied (once or serially) until a trial outcome was achieved. Notwithstanding, these limitations may have enhanced the generalizability of our findings to diverse ICUs and patients, many of whom likely had a high pretest probability for passing an initial SBT.^80^ Second, summary estimates for extubation outcome were qualitatively similar, but those for SBT outcome (pass or fail) varied across trials due to a single outlier trial. Third, despite finding significant differences in extubation outcome favoring PS (vs T-piece) SBTs, a subgroup analysis comparing SBT techniques with (vs without) inspiratory assistance did not identify credible subgroup effects favoring SBTs conducted with inspiratory assistance. We postulate that this may reflect a dilution phenomenon or a decrease in summary estimates by incorporation of trials that compared a lower amount of support (especially inspiratory) of SBT comparators compared with PS vs T-piece, which maximized differences in the support provided during SBTs. Fourth, few trials evaluated SBTs in children.^40,55,58^ Fifth, summary estimates were limited by heterogeneous reporting of weaning and extubation outcomes. Sixth, despite conduct of several subgroup analyses, we were not able to elucidate the optimal method to conduct an SBT in specific populations or using specific SBT techniques. Finally, the implications of the findings for patients with low pretest probability of SBT and extubation success and for patients who fail an initial SBT remain uncertain.

## Conclusions

This systematic review and meta-analysis found that critically ill adults or children undergoing PS compared with T-piece SBTs were more likely to pass an SBT and to be extubated successfully. Pressure support SBTs were not associated with increased risk of reintubation. Future investigations should include these findings and compare SBT techniques that maximize differences in inspiratory support.

## Notes

Supplement 1. **eAppendix.** Search Strategy for Each Database Searched **eFigure.** Risk of Bias of the Included Trials **eTable 1.** Summary of Findings Table—PS vs T-Piece SBTs on SBT Outcome **eTable 2.** Summary of Findings Table—PS vs T-Piece SBTs on Extubation Outcome **eTable 3.** Summary Estimates for Secondary Outcomes **eTable 4.** Subgroup Analyses

Supplement 2.
Data Sharing Statement
