# Supplementary material for: Spontaneous Breathing Trial Techniques for Extubating Adults and Children Who Are Critically Ill: A Systematic Review and Meta-Analysis
Source: JAMA Netw Open. 2024 Feb 23;7(2):e2356794. doi: 10.1001/jamanetworkopen.2023.56794 (PMC10891471; doi:10.1001/jamanetworkopen.2023.56794)
Supplement: Supplement 1. — eAppendix. Search Strategy for Each Database Searched eFigure. Risk of Bias of the Included Trials eTable 1. Summary of Findings Table—PS vs T-Piece SBTs on SBT Outcome eTable 2. Summary of Findings Table—PS vs T-Piece SBTs on Extubation Outcome eTable 3. Summary Estimates for Secondary Outcomes eTable 4. Subgroup Analyses [file jamanetwopen-e2356794-s001.pdf]

Supplemental Online Content

Burns KEA, Khan J, Phoophiboon V, et al. Spontaneous breathing trial techniques for extubating adults and children who are critically ill: a systematic review and meta-analysis. *JAMA Netw Open*. 2024;7(2):e2356794. doi:10.1001/jamanetworkopen.2023.56794

eAppendix. Search Strategy for Each Database Searched

eFigure. Risk of Bias of the Included Trials

eTable 1. Summary of Findings Table—PS vs T-Piece SBTs on SBT Outcome

eTable 2. Summary of Findings Table—PS vs T-Piece SBTs on Extubation Outcome

eTable 3. Summary Estimates for Secondary Outcomes

eTable 4. Subgroup Analyses

This supplemental material has been provided by the authors to give readers additional information about their work.

© 2024 Burns KEA et al. *JAMA Network Open*.

eAppendix. Search Strategy for Each Database Searched

Database: Ovid MEDLINE(R) In-Process & Other Non-Indexed Citations and Ovid MEDLINE(R) <1946 to Present>

Search Strategy:
----
1 artificial respiration.mp.
2 exp Respiratory Insufficiency/
3 (breath$ or respir$).mp. and insufficien$.ti,ab. [mp=title, abstract, original title, name of substance word, subject heading word, keyword heading word, protocol supplementary concept word, rare disease supplementary concept word, unique identifier]
4 Continuous positive airway/
5 CPAP.ti,ab.
6 exp Critical Care/is, mt [Instrumentation, Methods]
7 exp Intensive Care Units/is, mt [Instrumentation, Methods]
8 intermittent positive-pressure breathing/
9 ((intubation adj5 airway) or intra?trach or trach$).mp. [mp=title, abstract, original title, name of substance word, subject heading word, keyword heading word, protocol supplementary concept word, rare disease supplementary concept word, unique identifier]
10 exp Intubation, Intratracheal/
11 exp Intermittent Positive-Pressure Ventilation/
12 intermittent positive-pressure ventilation.ti,ab.
13 exp Ventilators, Mechanical/
14 ventilat$.ti,ab.
15 or/1-14
16 spontaneous breathing.mp.
17 SBT.ti.
18 exp Ventilator Weaning/
19 exp Airway Extubation/
20 extubation.ti,ab.
21 ATC.ti.
22 automatic tube compensation.mp.
23 breath tests.mp. or exp Breath Tests/
24 exp Device Removal/
25 extubation.mp.
26 gradual pressure support.mp.
27 t-tube.mp.
28 T-piece.mp.
29 unassisted breath$.mp.
30 wean$.ti,ab.
31 exp "Work of Breathing"/ph [Physiology]
32 Zeep.mp.
33 (ZEEP or PEEP).ti,ab.
34 or/16-32
35 15 and 34
36 *Noninvasive Ventilation/
37 NIV.ti.
38 *Neonatology/
39 *Intensive Care Units, Neonatal/
40 *infant, newborn/
41 Intellivent.mp.
42 smartcare.mp.
43 35 not (or/36-42)
44 limit 43 to randomized controlled trial
45 randomized controlled trial.pt. or randomized.mp. or placebo.mp.

© 2024 Burns KEA et al. *JAMA Network Open*.

46   43 and (44 or 45)
47   remove duplicates from 46

Adding clinical trials
48   clinical trial.mp. or clinical trial.pt. or random:.mp. or tu.xs.
49   43 and 48

****

Database: EBM Reviews - Cochrane Database of Systematic Reviews <2005 to February 2023>, EBM Reviews - ACP Journal Club <1991 to February 2023>, EBM Reviews - Database of Abstracts of Reviews of Effects <1st Quarter 2023>, EBM Reviews - Cochrane Central Register of Controlled Trials <February 2023>, EBM Reviews - Cochrane Methodology Register <1st Quarter 2023>, EBM Reviews - Health Technology Assessment <1st Quarter 2023>, EBM Reviews - NHS Economic Evaluation Database <1st Quarter 2023>

Search Strategy:
----
1   artificial respiration.mp.
2   exp Respiratory Insufficiency/
3   (breath$ or respir$).mp. and insufficien$.ti,ab. [mp=ti, ab, tx, kw, ct, ot, sh, hw]
4   Continuous positive airway/
5   CPAP.ti,ab.
6   exp Critical Care/is, mt [Instrumentation, Methods]
7   exp Intensive Care Units/is, mt [Instrumentation, Methods]
8   intermittent positive-pressure breathing/
9   ((intubation adj5 airway) or intra?trach or trach$).mp. [mp=ti, ab, tx, kw, ct, ot, sh, hw]
10   exp Intubation, Intratracheal/
11   exp Intermittent Positive-Pressure Ventilation/
12   intermittent positive-pressure ventilation.ti,ab.
13   exp Ventilators, Mechanical/
14   ventilat$.ti,ab.
15   or/1-14
16   spontaneous breathing.mp.
17   SBT.ti.
18   exp Ventilator Weaning/
19   exp Airway Extubation/
20   extubation.ti,ab.
21   ATC.ti.
22   automatic tube compensation.mp.
23   breath tests.mp. or exp Breath Tests/
24   exp Device Removal/
25   extubation.mp.
26   gradual pressure support.mp.
27   t-tube.mp.
28   T-piece.mp.
29   unassisted breath$.mp.
30   wean$.ti,ab.
31   exp "Work of Breathing"/ph [Physiology]
32   Zeep.mp.
33   (ZEEP or PEEP).ti,ab.
34   or/16-32
35   15 and 34
36   *Noninvasive Ventilation/
37   NIV.ti. 38   *Neonatology/
39   *Intensive Care Units, Neonatal/

© 2024 Burns KEA et al. *JAMA Network Open*.

40 *infant, newborn/
41 Intellivent.mp.
42 smartcare.mp.
43 neonate.mp. or newborn.ti. [mp=ti, ot, ab, tx, kw, ct, sh, hw]
44 preterm infant.ti,ab.
45 33 or 36 or 37 or 38 or 39 or 40 or 41 or 42 or 43 or 44
46 35 not 45

****

Database: Embase <1980 to 2023 Week 4>

Search Strategy:
----
1 exp artificial ventilation/
2 artificial respiration.mp.
3 (breath$ or respir$).mp. and insufficien$.ti,ab.
4 exp positive end expiratory pressure/
5 ("continuous positive airway pressure" or CPAP).ti,ab.
6 exp intensive care/
7 exp intermittent positive pressure ventilation/
8 ((intubation adj5 airway) or intra?trach or trach$).ti,ab.
9 exp mechanical ventilator/
10 ventilat$.ti,ab.
11 1 or 2 or 3 or 4 or 5 or 6 or 7 or 8 or 9 or 10
12 spontaneous breathing.mp.
13 SBT.ti.
14 exp artificial ventilation/ and wean$.ti.
15 exp extubation/
16 extubation.ti,ab.
17 ATC.ti.
18 automatic tube compensation.mp.
19 exp breath analysis/
20 exp device removal/
21 gradual pressure support.mp.
22 t-tube.mp.
23 T-piece.mp.
24 unassisted breath$.mp.
25 weaning/
26 wean.mp. or weaning.ti,ab. [mp=title, abstract, heading word, drug trade name, original title, device manufacturer, drug manufacturer, device trade name, keyword]
27 exp lung function test/
28 (ZEEP or PEEP).ti,ab.
29 or/12-28
30 11 and 29
31 *noninvasive ventilation/
32 NIV.ti.
36 31 or 32 or 33 or 34 or 35
37 30 not 36
38 double-blind:.mp. or placebo:.tw. or blind:.tw.
39 37 and 38

© 2024 Burns KEA et al. *JAMA Network Open*.

eFigure. Risk of Bias of the Included Trials

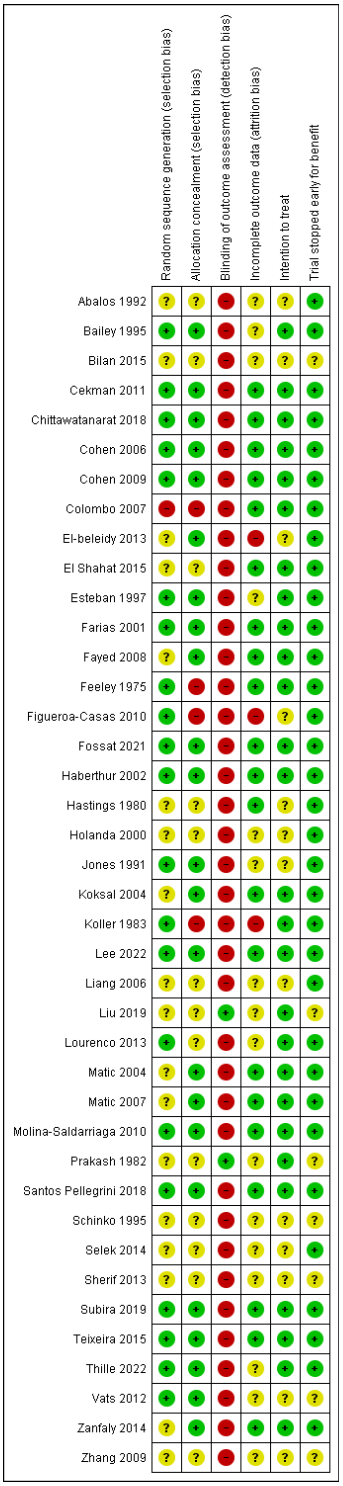

Figure Legend:

eFigure 1 depicts the risk of bias of the included trials. Green color indicates low risk of bias, yellow color indicates unclear risk of bias, and red color indicates high risk of bias.

© 2024 Burns KEA et al. *JAMA Network Open*.

eTable 1. Summary of Findings Table—PS vs T-Piece SBTs on SBT Outcome

PS compared to T-piece - SBT outcome (success) for weaning critically ill patients from invasive ventilation

Patient or population: patients with weaning critically ill patients from invasive ventilation
Settings:
Intervention: PS
Comparison: T-piece

Outcomes	Illustrative comparative risks* (95% CI)		Relative effect (95% CI)	No of Participants (studies)	Quality of the evidence (GRADE)	Comments
	Assumed risk	Corresponding risk				
	**T-piece**	**PS**				
**PS vs T-piece - SBT outcome (success)** Risk Ratio	**Study population**		**RR 1.04** (0.97 to 1.11)	4459 (14 studies)	⊕⊕⊖⊖ **low**[1,2]	
	**772 per 1000**	**803 per 1000** (749 to 857)				
	**Moderate**					
	**764 per 1000**	**795 per 1000** (741 to 848)				
	**960 per 1000**	**922 per 1000** (835 to 1000)				
	**960 per 1000**	**922 per 1000** (835 to 1000)				

*The basis for the **assumed risk** (e.g. the median control group risk across studies) is provided in footnotes. The **corresponding risk** (and its 95% confidence interval) is based on the assumed risk in the comparison group and the **relative effect** of the intervention (and its 95% CI).

CI: Confidence interval; **RR:** Risk ratio;

GRADE Working Group grades of evidence
High quality: Further research is very unlikely to change our confidence in the estimate of effect.
Moderate quality: Further research is likely to have an important impact on our confidence in the estimate of effect and may change the estimate.
Low quality: Further research is very likely to have an important impact on our confidence in the estimate of effect and is likely to change the estimate.
Very low quality: We are very uncertain about the estimate.

[1] Only one trial blinded outcomes assessment
[2] One trial accounted for heterogeneity. This trial was internally inconsistent as it had opposite findings for SBT outcome and extubation outcome. Removing this trial eliminated heterogeneity in SBT outcome

© 2024 Burns KEA et al. *JAMA Network Open*.

eTable 2. Summary of Findings Table—PS vs T-Piece SBTs on Extubation Outcome

PS compared to T-piece – extubation outcome for weaning critically ill patients from invasive ventilation

Patient or population: patients with weaning critically ill patients from invasive ventilation
Settings:
Intervention: PS
Comparison: T-piece

**Outcomes**	**Illustrative comparative risks* (95% CI)**		**Relative effect (95% CI)**	**No of Participants (studies)**	**Quality of the evidence (GRADE)**	**Comments**
	Assumed risk **T-piece**	Corresponding risk **PS**				
**PS vs T-piece - extubation outcome** RR	**Study population**		**RR 1.07** (1.04 to 1.1)	4462 (16 studies)	⊕⊕⊕⊖ **moderate**[1]	
	**721 per 1000**	**771 per 1000** (750 to 793)				
	**Moderate**					
	**720 per 1000**	**770 per 1000** (749 to 792)				

*The basis for the **assumed risk** (e.g. the median control group risk across studies) is provided in footnotes. The **corresponding risk** (and its 95% confidence interval) is based on the assumed risk in the comparison group and the **relative effect** of the intervention (and its 95% CI).

CI: Confidence interval; **RR:** Risk ratio;

GRADE Working Group grades of evidence
High quality: Further research is very unlikely to change our confidence in the estimate of effect.
Moderate quality: Further research is likely to have an important impact on our confidence in the estimate of effect and may change the estimate.
Low quality: Further research is very likely to have an important impact on our confidence in the estimate of effect and is likely to change the estimate.
Very low quality: We are very uncertain about the estimate.

[1] Only one trial reporting blinding of outcomes assessment

© 2024 Burns KEA et al. *JAMA Network Open*.

eTable 3. Summary Estimates for Secondary Outcomes

Comparison	Number of Trials (Patients)	Mean Difference (95% CI; p-value, I^2)
ICU Length of Stay (days)		
T-piece vs PS	9 (1754 vs 1721)	+0.16 (-0.26, +0.57; p=0.46; I^2=23%)
T-piece vs CPAP	2 (59 vs 52)	-3.63 (-10.52, +3.26; p=0.30; I^2=76%)
T-piece vs PAV+	1 (66 vs 48)	+0.50 (-2.69, +3.69; p=0.76; I^2=n/a)
T-piece vs HFNC	3 (197 vs 189)	+0.92 (-0.22, +2.05; p=0.11; I^2=0%)
T-piece vs ATC	1 (30 vs 30)	0.00 (-2.96, +2.96; p=1.00; I^2=n/a)
CPAP vs ATC	1 (30 vs 30)	+0.80 (-2.23, +3.83; p=0.60; I^2=n/a)
CPAP vs PS	1 (30 vs 30)	-0.30 (-2.23, +1.63; p=0.76; I^2=n/a)
PS vs PAV+	1 (46 vs 48)	+0.40 (-2.90, +3.70; p=0.81; I^2=n/a)
PS vs HFNC	1 (96 vs 82)	+1.00 (-2.09, +4.09; p=0.53; I^2=n/a)
PS vs ATC	2 (137 vs 125)	+0.73 (-1.03, +2.50; p=0.41; I^2=0%)
Hospital Length of Stay (days)		
T-piece vs PS	8 (1269 vs 1237)	-0.45 (-1.47, +0.58; p=0.39; I^2=0%)
T-piece vs CPAP	2 (59 vs 52)	+0.74 (-2.15, +3.63; p=0.61; I^2=0%)
T-piece vs PAV+	1 (66 vs 48)	+2.90 (-3.00, +8.80; p=0.34; I^2=n/a)
T-piece vs HFNC	3 (197 vs 189)	+1.46 (-1.42, +4.34; p=0.32; I^2=0%)
T-piece vs ATC	1 (30 vs 30)	-0.20 (-2.96, +2.56; p=0.89; I^2=n/a)
CPAP vs ATC	1 (30 vs 30)	-0.90 (-3.39, +1.59; p=0.48; I^2=n/a)
CPAP vs PS	1 (30 vs 30)	-1.10 (-3.59, +1.39; p=0.39; I^2=n/a)
PS vs PAV+	1 (46 vs 48)	+5.40 (-1.79, +12.59; p=0.14; I^2=n/a)
PS vs HFNC	1 (96 vs 82)	+1.00 (-2.44, +4.44; p=0.57; I^2=n/a)
PS vs ATC	2 (118 vs 108)	-0.27 (-1.85, +1.30; p=0.73; I^2=0%)
**Comparison**	**Number of Trials (Patients)**	**Risk Ratio (95% CI; p-value, I^2)**
Mortality (Longest Reported)		
T-piece vs PS	9 (1919 vs 1897)	1.11 (0.95, 1.30; p=0.18; I^2=0%)
T-piece vs CPAP	3 (67 vs 59)	0.55 (0.14, 2.08; p=0.38; I^2=35%)
T-piece vs PAV+	1 (69 vs 49)	0.71 (0.24, 2.07; p=0.53; I^2=n/a)
T-piece vs HFNC	2 (144 vs 136)	1.10 (0.68, 1.78; p=0.70; I^2=0%)
T-piece vs ATC	1 (25 vs 25)	0.50 (0.05, 5.17; p=0.56; I^2=n/a)
CPAP vs ATC/CPAP	1 (15 vs 15)	1.50 (0.29, 7.73; p=0.63; I^2=n/a)
PS vs PAV+	1 (47 vs 49)	1.04 (0.36, 3.00; p=0.94; I^2=n/a)
PS vs HFNC	1 (96 vs 82)	0.85 (0.05, 13.44; p=0.91; I^2=n/a)
PS vs ATC	1 (88 vs 78)	1.48 (0.77, 2.82; p=0.24; I^2=n/a)
Use of NIV After Extubation		
T-piece vs PS	3 (1132 vs 1106)	0.95 (0.89, 1.02; p=0.17; I^2=0%)
T-piece vs PAV+	1 (69 vs 49)	1.26 (0.61, 2.62; p=0.53; I^2=n/a)
T-piece vs HFNC	2 (107 vs 107)	0.98 (0.65, 1.47; p=0.92; I^2=0%)
CPAP vs ATC/CPAP	3 (123 vs 124)	1.87 (0.95, 3.71; p=0.07; I^2=0%)
PS vs PAV+	1 (47 vs 49)	1.27 (0.58, 2.79; p=0.55; I^2=n/a)
PS vs ATC	1 (88 vs 78)	0.83 (0.64, 1.08; p=0.17; I^2=n/a)
Use of HFNC After Extubation		
T-piece vs PS	2 (1063 vs 1059)	1.09 (0.75, 1.57; p=0.66; I^2=48%)

© 2024 Burns KEA et al. *JAMA Network Open*.

T-piece vs HFNC	2 (107 vs 107)	0.89 (0.66, 1.20; p=0.43; I^2=25%)
Tracheostomy		
T-piece vs PS	5 (1082 vs 1047)	1.22 (0.90, 1.67; p=0.20; I^2=0%)
T-piece vs PAV+	1 (69 vs 49)	0.88 (0.22, 3.46; p=0.85; I^2=n/a)
T-piece vs HFNC	1 (90 vs 82)	1.84 (0.16, 20.69; p=0.62; I^2=n/a)
PS vs PAV+	1 (47 vs 49)	1.82 (0.57, 5.83; p=0.31; I^2=n/a)
PS vs HFNC	1 (96 vs 82)	1.71 (0.16, 18.50; p=0.66; I^2=n/a)

Legend

ICU = intensive care unit; PS = pressure support, CPAP = continuous positive airway pressure, PAV+ = proportional assist ventilation plus; HFNC = high flow nasal cannulae, ATC = automatic tube compensation; NIV=noninvasive ventilation, CI = confidence interval

© 2024 Burns KEA et al. *JAMA Network Open*.

eTable 4. Subgroup Analyses

Subgroup comparison	Outcome	Summary Estimate[a] p-value, heterogeneity; [n, no of trials]	Summary Estimate[b] p-value, heterogeneity; [n, no of trials]	Subgroup Differences p-value, heterogeneity (interaction)
**PS vs T-piece Trials**				
COPD[a] vs. non-COPD[b]	SBT Outcome	RR 1.00, 95% CI 0.87–1.14; p = 0.96, I^2 = 0%; [n=326; 2 trials]	RR 1.04, 95% CI 0.97–1.12; p = 0.29, I^2 = 77%; [n=4,133, 12 trials]	0.59, I^2=0%
	Extubation Outcome	RR 1.06, 95% CI 0.89–1.25; p = 0.52, not estimable; [n=190, 1 trial]	RR 1.07, 95% CI 1.04-1.10; p<0.0001, I^2 = 0%; [n=4,272, 15 trials]	0.92, I^2=0%
PEEP ($\leq$ 5 cm H_2O[a] vs. $\geq$ 6 cm H_2O[b]) level during SBTs	SBT Outcome	RR 1.00, 95% CI 0.87-1.14; p=0.95, I^2 = 91%; [n=2,959, 5 trials]	RR 1.04, 95% CI 0.86–1.25; p = 0.69, I^2 = 46%; [n=302, 2 trials]	0.72, I^2=0%
	Extubation Outcome	RR 1.08, 95% CI 1.04–1.12; p<0.0001, I^2=0%; [n=3,327, 8 trials]	RR 1.03, 95% CI 0.97 - 1.09; p=0.36, I^2=0%; [n=298, 4 trials]	0.19, I^2=42.2%
PS ($\leq$ 8 cm H_2O[a] vs. $\geq$ 9 cm H_2O[b]) utilized during SBTs	SBT Outcome	RR 1.05, 95% CI 0.96–1.14; p = 0.30, I^2 =81%; [n=3,848, 10 trials]	RR 1.01, 95% CI 0.93–1.09; p = 0.87, I^2 =0%; [n=475, 3 trials]	0.51, I^2 =0%
	Extubation Outcome	RR 1.07, 95% CI 1.04–1.10; p <0.0001, I^2 =0%; [n=3,947, 13 trials]	RR 1.01, 95% CI 0.93–1.11; p = 0.75, I^2 =0%; [n=475, 3 trials]	0.27; I^2 =19.4%
**All SBT trials**				
Inspiratory assistance[a] vs. no inspiratory assistance[b] during SBTs	SBT Outcome	RR 1.04, 95% CI 0.99–1.09; p=0.15, I^2 =68%; [n=5,212, 23 trials]	RR 1.02, 95% CI 0.98–1.06; p=0.37, I^2 =44%; [n=823, 11 trials]	0.63; I^2 =0%
	Extubation Outcome	RR 1.05, 95% CI 1.02–1.09; p=0.004, I^2 =33%; [n=4,844, 21 trials]	RR 1.06, 95% CI 1.00–1.12; p=0.06, I^2 =23%; [n=682, 8 trials]	0.89; I^2 =0%

Legend

COPD = chronic obstructive pulmonary disease, SBT = spontaneous breathing trial, RR = relative risk/risk ratio, CI = confidence interval, PS = pressure support, PEEP = positive end-expiratory pressure.

© 2024 Burns KEA et al. *JAMA Network Open*.
