# Supplementary material for: Spontaneous Breathing Trial Techniques for Extubating Adults and Children Who Are Critically Ill: A Systematic Review and Meta-Analysis
Source: JAMA Netw Open. 2024 Feb 23;7(2):e2356794. doi: 10.1001/jamanetworkopen.2023.56794 (PMC10891471; doi:10.1001/jamanetworkopen.2023.56794)
Supplement: Supplement 2. — Data Sharing Statement [file jamanetwopen-e2356794-s002.pdf]

Data Sharing Statement

Burns. Spontaneous Breathing Trial Techniques for Extubating Adults and Children Who Are Critically Ill. *JAMA Netw Open*. Published February 23, 2024.
doi:10.1001/jamanetworkopen.2023.56794

Data
Data available: Yes
Data types: Data (not involving human participants)
How to access data: Raw data are available upon written request to Dr. Burns.
When available: With publication

Supporting Documents
Document types: None

Additional Information
Who can access the data: Raw data are available upon written request to Dr. Burns.
Types of analyses: RevMan files
Mechanisms of data availability: written request
Any additional restrictions: None
